# Mitofilin Mitigates Myocardial Damage in Acute Myocardial Infarction by Regulating Pyroptosis of Cardiomyocytes

**DOI:** 10.3389/fcvm.2022.823591

**Published:** 2022-05-02

**Authors:** Min Ma, Shi-chu Liang, Kai-yue Diao, Qin Wang, Yong He

**Affiliations:** ^1^Department of Cardiology, West China Hospital, Sichuan University, Chengdu, China; ^2^Department of Cardiology, The Sixth People's Hospital of Chengdu, Chengdu, China; ^3^Department of Radiology, State Key Laboratory of Biotherapy, West China Hospital, Sichuan University, Chengdu, China

**Keywords:** myocardial infarction, pyroptosis, mitofilin, PI3K/AKT, cardiomyocytes

## Abstract

**Background:**

Acute myocardial infarction (AMI) can lead to sudden cardiac death after prolonged ischemia or heart failure (HF) and impaired left ventricular pump function. However, the underlying mechanism remains largely unknown. The purpose of this study was to investigate the role of mitofilin in alleviating AMI.

**Methods:**

Recombinant adenoviral vectors for mitofilin overexpression or mitofilin knockdown were constructed, respectively. A mouse AMI model was established and the effect of mitofilin on myocardial pyroptosis was examined by detecting the lactate dehydrogenase (LDH) level and inflammatory factors. Moreover, a cellular model of AMI was established by treating cardiomyocytes with hypoxia/reoxygenation (H/R). An enzyme-linked immunosorbent assay (ELISA) and a western blot analysis were used to detect the effect of mitofilin knockdown on the expression of pyroptosis-related factors. Furthermore, the regulatory role of mitofilin in PI3K/AKT pathway was evaluated by the western blot and PI3K inhibitor.

**Results:**

Mitofilin was downregulated in the heart tissue of the AMI mice and H/R induced cardiomyocytes. The overexpression of mitofilin significantly alleviated AMI and reduced pyroptosis-related factors. Meanwhile, in cardiomyocytes, mitofilin knockdown aggravated cellular damages by promoting pyroptosis. Further analysis showed that the anti-pyroptotic effect of mitofilin was dependent on the activation of the PI3K/AKT signaling pathway.

**Conclusions:**

Our study suggests that mitofilin regulates pyroptosis through the PI3K/AKT signaling pathway in cardiomyocytes to ameliorate AMI, which may serve as a therapeutic strategy for the management of AMI.

## Introduction

Acute myocardial infarction (AMI) usually results from an abrupt coronary occlusion that leads to ischemia and necrosis of the myocardium in the corresponding perfusion area. Prolonged ischemia can develop into heart failure (HF) and impair left ventricular pump function, which eventually results in sudden cardiac death (1, 2). AMI is one of the leading causes of death worldwide and is prevalent in the middle-aged and older adult populations (3). Intense inflammatory responses have been observed during the occurrence of AMI, which have been recognized as major catastrophic events accounting for excessive injury and dysregulation of ventricular remodeling (4, 5).

Pyroptosis is a highly inflammatory form of programmed cell death frequently observed under inflammatory conditions (6). It is characterized by cell swelling and membrane ruptures, leading to the release of cellular contents and the activation of a strong inflammatory response (7). Pyroptosis is an important natural immune defense in the body and plays an important role in fighting infections (8). Many studies have implicated pyroptosis in the pathogenesis of a variety of cardiovascular diseases (CVDs), such as AMI (9). However, the inducing factors and regulatory mechanisms of pyroptosis in AMI remain to be elucidated.

Mitofilin is a ubiquitously expressed mitochondrial protein present in the mitochondrial inner membrane (10). Mitofilin was originally identified as a myocardial mitochondrial protein because it is preferentially expressed in the heart tissues (11). It has been shown that mitofilin knockdown can lead to the degradation and functional impairment of mitochondria in cultured H9C2 cells and HEK 293 cells, which can eventually lead to apoptosis through the PARP-AIF mechanism, and the overexpression of mitofilin can protect H9C2 cardiomyocytes from oxidative damage caused by H_2_O_2_ (12). Furthermore, it has been reported the knockdown of mitofilin leads to the disruption of mitochondrial morphology and networks and the initiation of apoptosis (13). However, the potential role of mitofilin in regulating myocardial injury caused by AMI is largely unknown.

In this study, we established a mouse model of AMI with adenovirus-mediated mitofilin overexpression and examined the role of mitofilin in cellular damages and pyroptosis in AMI. In the cellular model of hypoxia/reoxygenation (H/R) induced H9C2 cardiomyocytes, the effects of mitofilin knockdown and overexpression on pyroptosis were further evaluated. We further demonstrated that the activation of the PI3K/AKT signaling pathway was indispensable in the anti-pyroptotic role of mitofilin.

## Materials and Methods

### Establishment of the Myocardial Infarction Mouse Model

The male C57BL/6 mice (about 8 weeks, 22–25 g) were housed at 23 ± 2°C under a cycle of 12/12 h light/ dark condition with free access to food and water. The AMI model was established as described previously (14). The 2,2,2-tribromoethanol (200 mg/kg; Sigma, St. Louis, MO, USA) was intraperitoneally injected to anesthetize animals. In the AMI group, a left thoracotomy was performed with a small incision at the third and fourth intercostal space, and a 7-0 prolene suture (Ethicon, Inc., Somerville, NJ, USA) was applied to ligate the left anterior descending branch (LAD). Mice in the sham group underwent the same procedure without stitches. The mice were sacrificed by cervical dislocation at 12 h after myocardial infarction (MI). All operations were performed under aseptic conditions. The procedures for experiments and animal care were approved by the West China Hospital (Approval Number: 2020020A) and conformed to the Guide for the Care and Use of Laboratory Animals produced by the National Institutes of Health.

### Detection of Myocardial Infarct Area

Evans Blue and 2,3,5triphenyl tetrazolium chloride (TTC) staining were used to evaluate the infarct area of ischemia/reperfusion (I/R) injury according to previous reports (15). Mice subjected to LAD ligation for 30 min and reperfusion for 4 h were intravenously injected with 2% Evans Blue *via* the jugular vein. Then, the hearts of rats were washed with PBS and cut into 4 μm thick slices from the apex to the base. After incubation with 2% TTC for 20 min, the sections were divided into 3 areas, namely, non-ischemic area (blue), risk area (AAR area, dark red), and infarct area (IA area, pale white). Sections of the cardiac papillary muscle plane were captured under a microscope and quantified using Image-Pro Plus 6.0 (Media Cybernetics). The percentage of infarct area was calculated by the following formula: IA/total left ventricle (LV) ×100%.

### Determination of Necrotic Cell Death of Cardiomyocytes

The cellular malondialdehyde (MDA) level and lactate dehydrogenase (LDH) level were assessed after the indicated treatments by using commercially available biochemical kits (Beijing KEMEI DONGYA Biotechnology Ltd.). The procedures were performed according to the manufacturer's instructions.

### Enzyme-Linked Immunosorbent Assay

The concentration of interleukin (IL)-1β and IL-18 in the serum of MI mice and the supernatant of cell cultures were detected by commercial ELISA kits (Nanjing Jiancheng Bioengineering Institute, Nanjing, China) according to the manufacturer's instructions (16). The absorbance at 450 nm of each well was measured using a Synergy H1 microplate reader (Winooski, Vermont, USA).

### Western Blotting Assay

Proteins were extracted from mouse heart tissue and cardiomyocytes using a Radio Immunoprecipitation Assay (RIPA) assay buffer containing protease inhibitors (Solarbio, Beijing, China) and analyzed using the western blot. Cells or tissues were suspended in RIPA buffer, lysed on ice for 15 min and then centrifuged at 14,000 rpm for 10 min. The supernatant containing total protein lysate was quantified using a BCA kit (Beyotime, Shanghai, China). Then, 10 μg of protein was used for sodium dodecyl-sulfate polyacrylamide gel electrophoresis (SDS-PAGE) in 10% protein gel and then transferred to nitrocellulose membranes and blocked with 5% bovine serum albumin (BSA) at room temperature for 2 h. Thereafter, the membranes were incubated overnight at 4°C with antibodies against p-PI3K, t-PI3K, p-AKT, t-AKT, nucleotide-binding oligomerization domain-like receptor pyrin domain-containing 3 (NLRP3), cleaved caspase-1, pro-caspase-1, apoptosis-associated speck-like protein containing (ASC), and glyceraldehyde-3-phosphate dehydrogenase (GAPDH) (dilution, 1:1,000, all antibodies are from Abcam). After washing with TBST buffer, membranes were incubated with the horseradish peroxidase-conjugated secondary antibody (1:3,000; Cell Signaling Technologies, MA, USA) at room temperature for 1 h. The protein bands were visualized using an enhanced chemiluminescence (ECL) system (Thermo Fisher Scientific, Inc).

### H9C2 Cell Culture and Treatment

Rat heart-derived H9C2 cells were purchased from ATCC and cultured using DMEM medium (Hyclone, Logan, UT, USA) containing 100 IU/ml of penicillin–streptomycin (Beyotime, Shanghai, China) and 10% fetal bovine serum (Hyclone, Logan, UT, USA) at 37°C in a 5% CO_2_ incubator. For H/R treatment, H9C2 cells were subjected to 16 h of hypoxia (O_2_:N_2_:CO_2_, 1:94:5) followed by 2 h of reoxygenation. To inhibit the activity of caspase-1, cells were treated with AC-YVAD-CMK (10 μg/ml, InvivoGen, San Diego, CA, USA). To suppress the activity of PI3K, cells were treated with 5 μM GDC-0941 (MedChemExpress, Monmouth Junction, NJ 08852, USA). All experiments were performed in 12-well plates, and cells were seeded at a density of 0.1 × 10^6^ cells/well. Cells were collected for further analysis 24 h after indicated treatments.

### Adenovirus Production and Transduction Into Cardiomyocytes *in vivo*

A recombinant adenoviral vector encoding mitofilin cDNA and its negative control were obtained from GeneChem (Montreal, Quebec Canada). The vectors were transfected into 293ET cells together with the AdMax packaging system (GeneChem) using polyethylenimine (PEI, Sigma, Germany). Then, 2 days after transfection, viruses in the supernatant were collected by centrifuging to remove cell debris. The virus was concentrated using the Fast Trap Adenovirus Purification and Concentration Kit (Sigma, Germany) and routinely titrated to 1.0 × 10^10^ infectious units (IFU)/ml in H92C cells using the Adeno-X™ Rapid Titer Kit (Takara, Dalian, China). For viral transduction in the *in vivo* study, 100 μl of adenovirus solutions were intramyocardially injected evenly into five sites in the heart, and MI was induced 48 h after viral administration.

### Propidium Iodide Staining for Membrane Integrity

To determine the cell membrane integrity, cells after the indicated treatment were stained with 5 μg/ml propidium iodide (PI) and 1 μg/ml Hoechst 33342 (Thermo Fisher Scientific, WA, USA) for 15 min in the medium. After that, cells were washed two times with a fresh medium and the morphology and stained cells were observed under the EVOS Cell Imaging System (Thermo Fisher Scientific, WA, USA).

### Quantitative Reverse Transcription PCR Analysis

Trizol reagent (Thermo Fisher Scientific) was used to extract RNA from tissues and cells according to the instructions. The extracted total RNA was dissolved in diethylpyrocarbonate (DEPC) water and the concentration was determined with NanoDorp. Then, 1 μg of total RNA was reverse transcribed into cDNA using the RevertAid First Strand cDNA Synthesis Kit (Thermo Fisher Scientific) and quantified in a 7500 Real-Time PCR System (Applied Biosystems, CA, USA) using the SYBR premix EX TAQ II kit (Takara, Dalian, China). The 2–ΔΔCt method was used to analyze the relative expression level with GAPDH as the internal reference. All primer sequences were synthesized by Shanghai Sangon Biotechnology Co., Ltd. (Shanghai, China): GAPDH: F- TGGCCT TCCGTGT TCCTAC and R- GAGTTGC TGTTGAAG TCGCA; and Mitofilin: F- CGACCA TGCCGTAG ATACTCC and R- CACCAA CGAACAA AAGGCCA.

### Statistical Analysis

The data were expressed as means ± SD. Student's *t*-test was used for the comparison of the two groups. Differences among multiple groups were analyzed by one-way ANOVA, with Tukey's *post-hoc* test for pairwise comparison. A value of *p* < 0.05 was set as statistical significance. All statistical analyses were performed by GraphPad Prim 5 (GraphPad Software, La Jolla, CA, USA).

## Results

### Downregulation of Mitofilin Expression in AMI Mice

**Figure 1:**
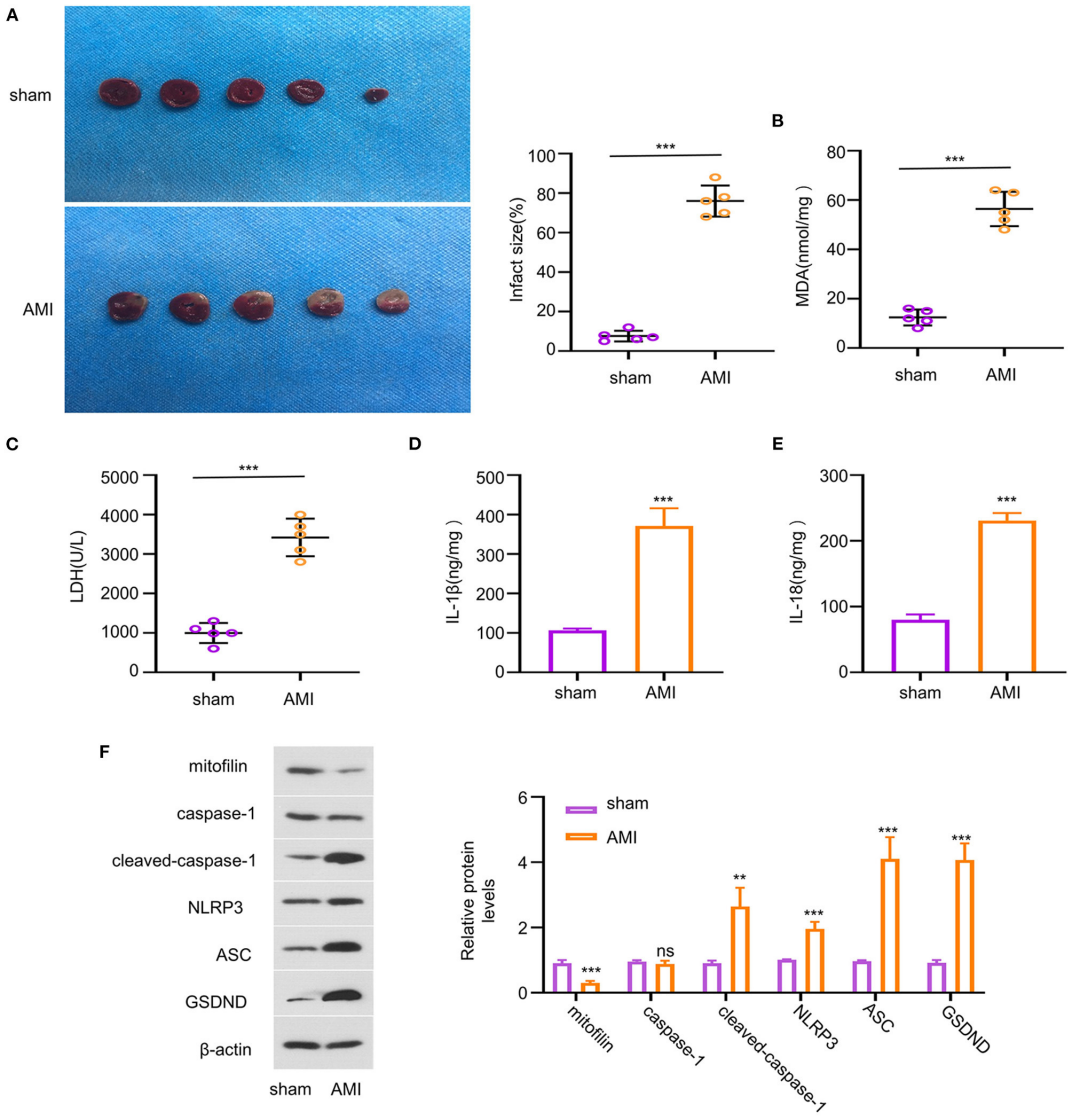
Reduced mitofilin expression is associated with the upregulation of pyroptosis-related factors in an acute myocardial infarction (AMI) model. **(A)** The percentage of myocardial infarction (MI) area increased in the AMI group. **(B,C)** The malondialdehyde (MDA) level and the lactate dehydrogenase (LDH) level were increased upon AMI induction. **(D,E)** The levels of interleukin (IL)-1β and IL-18 were increased in the AMI group. **(F)** Mitofilin protein levels were significantly decreased in the AMI group, while the protein levels of cleaved-caspase-1, nucleotide-binding oligomerization domain-like receptor pyrin domain-containing 3 (NLRP3), apoptosis-associated speck-like protein containing (ASC), Gasdermin D (GSDND) were increased. **p < 0.01 and ***p < 0.001. ns: not significant.

To evaluate the potential role of mitofilin in AMI, we investigated the expression level of mitofilin in a mouse model of AMI. The results showed that the percentage of MI area in the AMI group was statistically higher than that in the sham group (Figure 1A). We next measured the level of oxidative stress (MDA) and cell death (LDH release) in cardiac tissues, which revealed that AMI induction caused a significant increase in both MDA and LDH levels (Figures 1B,C). Additionally, the concentrations of pro-inflammatory factors related to pyroptosis (IL-1β and IL-18) were also increased in the AMI group (Figures 1D,E). Consistently, the protein levels of cleaved-caspase-1, NLRP3, ASC (PYD and CARD domain-containing proteins), and Gasdermin D (GSDND) were also upregulated in the AMI group (Figure 1F). However, the expression level of mitofilin was markedly reduced in the heart tissues of AMI compared with the sham group (Figure 1F). These results indicate that AMI induces pyroptosis in heart tissues, which is associated with a decrease in mitofilin expression.

### Overexpression of Mitofilin Ameliorates Pyroptosis in AMI

**Figure 2:**
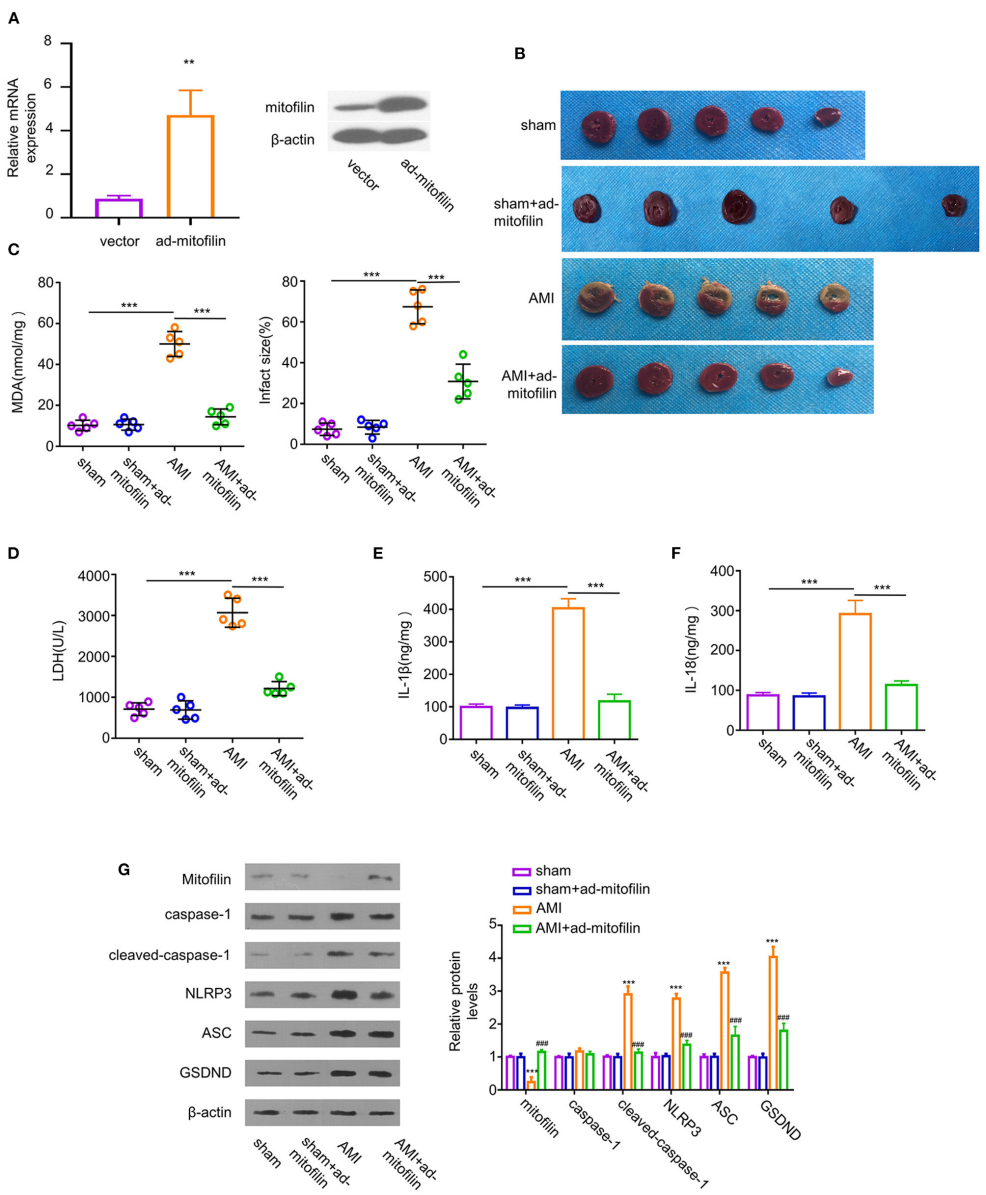
Overexpression of mitofilin alleviates AMI. **(A)** The administration of adenovirus carrying mitofilin (ad-mitofilin) significantly increased the expression level of mitofilin in the myocardium. **(B)** The area of MI was partially reduced after the administration of ad-mitofilin. **(C,D)** MDA levels and LDH levels were partially reduced after the administration of ad-mitofilin. **(E,F)** IL-1β and IL-18 levels were partially reduced after the administration of ad-mitofilin. **(G)** After the administration of ad-mitofilin, the mitofilin protein level was significantly increased upon AMI induction, while the protein levels of cleaved- caspase-1, NLRP3, ASC, and GSDND protein levels were partially decreased. Sham vs AMI: **P < 0.01 and ***P < 0.001. AMI vs AMI+ad-mitofilin: ### *P* < 0.001.

To assess the role of mitofilin in the pyroptosis of the AMI model, we overexpressed mitofilin in AMI rats by infecting cardiomyocytes with adenovirus carrying the mitofilin gene (ad-mitofilin). The results showed that mitofilin was significantly upregulated at protein and mRNA levels in the myocardium after the administration of ad-mitofilin (Figure 2A). Compared with the sham group, the percentage of MI area in the AMI group was higher, while the area of MI was partially reduced after the administration of ad-mitofilin (Figure 2B). We next measured the oxidative stress, cell death, as and pro-inflammatory cytokines in the above groups. The increased levels of MDA, LDH, IL-1β, and IL-18 induced in the AMI group were effectively reduced after the administration of ad-mitofilin (Figures 2C–F). In addition, the overexpression of mitofilin significantly decreased the protein levels of cleaved caspase-1, NLRP3, ASC, and GSDND (Figure 2G). Therefore, mitofilin overexpression shows a protective effect against pyroptosis in the AMI model.

### Knockdown of Mitofilin Exacerbates H/R-Induced Pyroptosis in Cardiomyocytes

**Figure 3:**
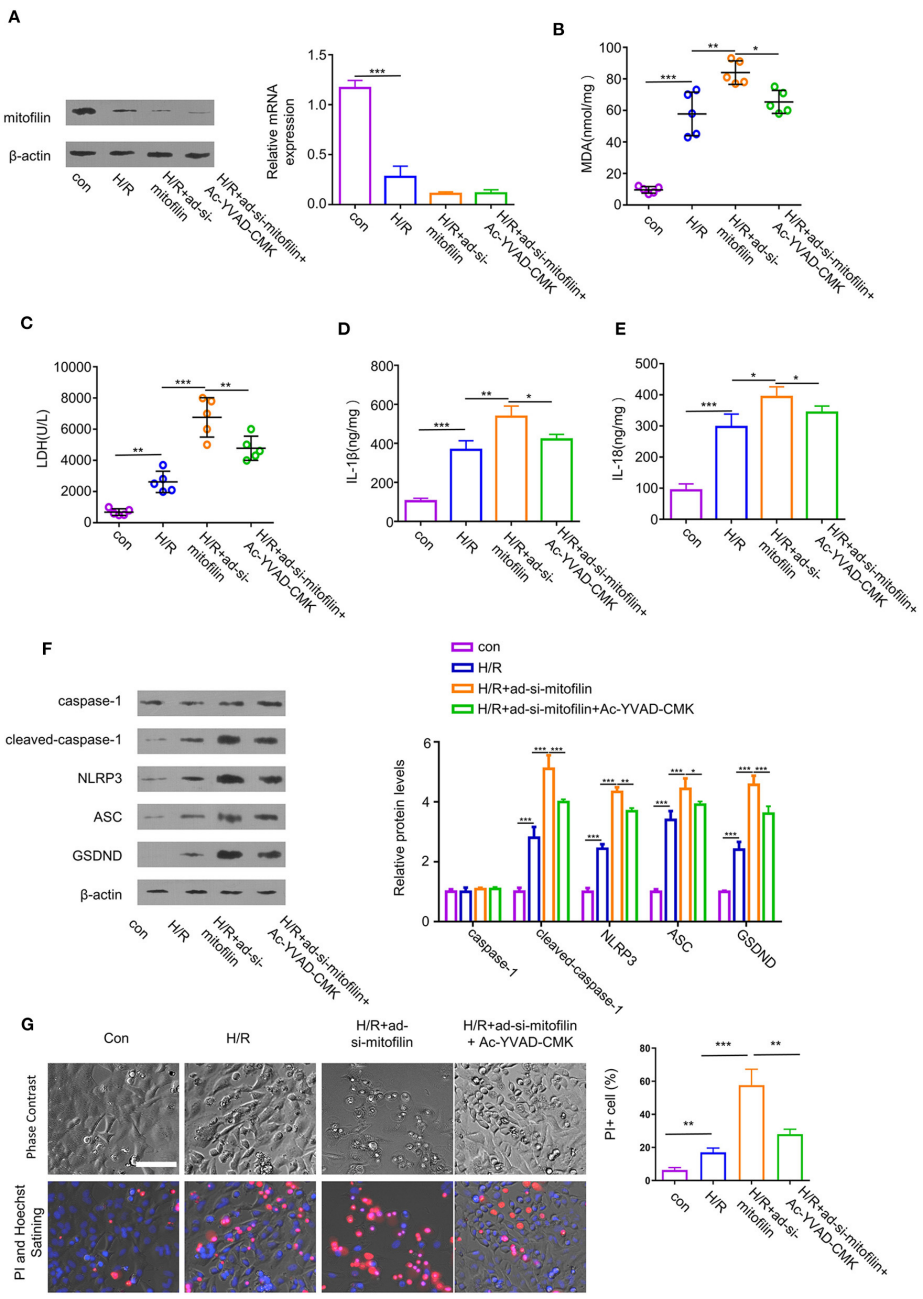
Mitofilin knockdown exacerbates hypoxia/reoxygenation (H/R)-induced cell damage of H9C2 by regulating pyroptosis. **(A)** The expression level of mitofilin was strongly reduced after the administration of ad-si-mitofilin. **(B,C)** H/R induced the increase of MDA level and LDH level in H9C2 cells, while levels were further increased after the administration of ad-si-mitofilin. The presence of AC-YVAD-CMK (an inhibitor for caspase-1) suppressed the effect of mitofilin knockdown. **(D,E)** H/R induced the increase of IL-1β and IL-18 levels in H9C2 cells, which were further increased after the mitofilin knockdown. The presence of AC-YVAD-CMK suppressed the effect of mitofilin knockdown. **(F)** Mitofilin knockdown increased the protein levels of cleaved-caspase-1, NLRP3, ASC, and GSDND in H9C2 cells upon H/R induction, which was rescued by AC-YVAD-CMK. **(G)** H/R induction promoted cell death by increasing the percentage of cells with impaired membrane integrity [revealed by propidium iodide (PI) staining]. Mitofilin knockdown exacerbated cell death (with typical morphology of cell swelling and membrane rupture). AC-YVAD-CMK suppressed the effect of mitofilin knockdown. *P < 0.05, **P < 0.01, and ***P < 0.001.

To further evaluate the indispensability of mitofilin in pyroptosis in cardiomyocytes, we established an H/R-induced cell damage model in cardiomyocytes. In addition, we transduced the cells with an adenovirus carrying shRNA for mitofilin silencing (ad-si-mitofilin) to investigate the effect of mitofilin knockdown on H/R induced cardiomyocytes injury. H/R treatment induced a decrease in mitofilin protein level in H9C2 cells, which was further reduced after the administration of ad-si-mitofilin (Figure 3A). As expected, the levels of MDA, LDH, IL-1β, and IL-18 in H9C2 cells were increased after H/R induction, which were further increased after mitofilin knockdown (Figures 3B–E). Mitofilin knockdown also promoted the protein levels of cleaved-caspase-1, NLRP3, ASC, and GSDND in H/R-induced H9C2 cells (Figure 3F). To corroborate that the observed effects rely on caspase-1 dependent pyroptosis, we applied AC-YVAD-CMK (an inhibitor for caspase-1) after mitofilin knockdown. The data showed that the administration of AC-YVAD-CMK mitigated oxidative stress and inflammatory cytokines, as well as partially reduced the levels of pyroptosis-related proteins (Figures 3B–F). Furthermore, we evaluated the cell morphology and membrane integrity by PI staining using a microscope. H/R treatment significantly increased the percentage of cells staining positive for PI, and mitofilin knockdown considerably increased the percentage of PI-positive cells, which displayed typical cell swelling and membrane rupture (Figure 3G). The presence of AC-YVAD-CMK could partially rescue this effect. Together, these data suggest that mitofilin is indispensable for protecting H/R-induced pyroptosis in cardiomyocytes.

### Mitofilin Regulates PI3K/AKT Pathway in Cardiomyocytes

**Figure 4:**
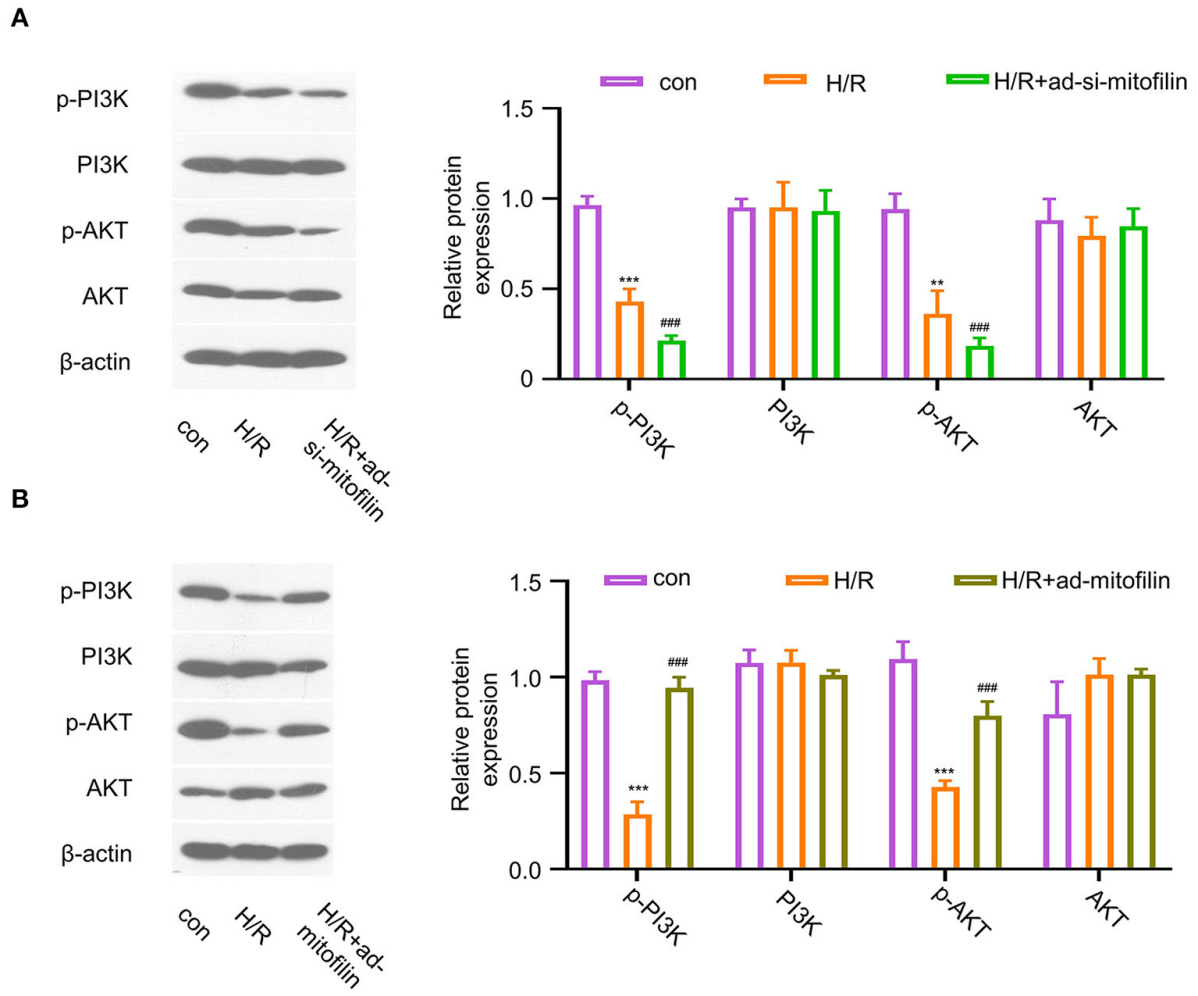
Mitofilin regulates the PI3K/AKT signaling pathway. **(A)** The levels of p-PI3K and p-AKT in H9C2 cells were decreased after H/R induction, which were further decreased after the mitofilin knockdown. **(B)** The levels of p-PI3K and p-AKT in H9C2 cells were rescued after mitofilin overexpression. Con vs H/R: **P < 0.01 and *** *P* < 0.001. H/R vs H/R+ad-si-mitofilin or ad-mitofilin: ### *P* < 0.001.

To study the possible molecular mechanisms of mitofilin in alleviating AMI and pyroptosis, we evaluated the activation status of the PI3K/AKT pathway. The results showed that, in H9C2 cells, H/R induction remarkably suppressed the PI3K/AKT pathway by reducing the phosphorylation levels of p-PI3K and p-AKT, and the levels of p-PI3K and p-AKT were further decreased after mitofilin knockdown (Figure 4A). In contrast, the overexpression of mitofilin rescued the levels of p-PI3K and p-AKT upon H/R induction (Figure 4B). These data indicate that PI3K/AKT pathway is involved in the regulation of pyroptosis by mitofilin.

### The Anti-pyroptotic Effect of Mitofilin Is Dependent on PI3K Activity

**Figure 5:**
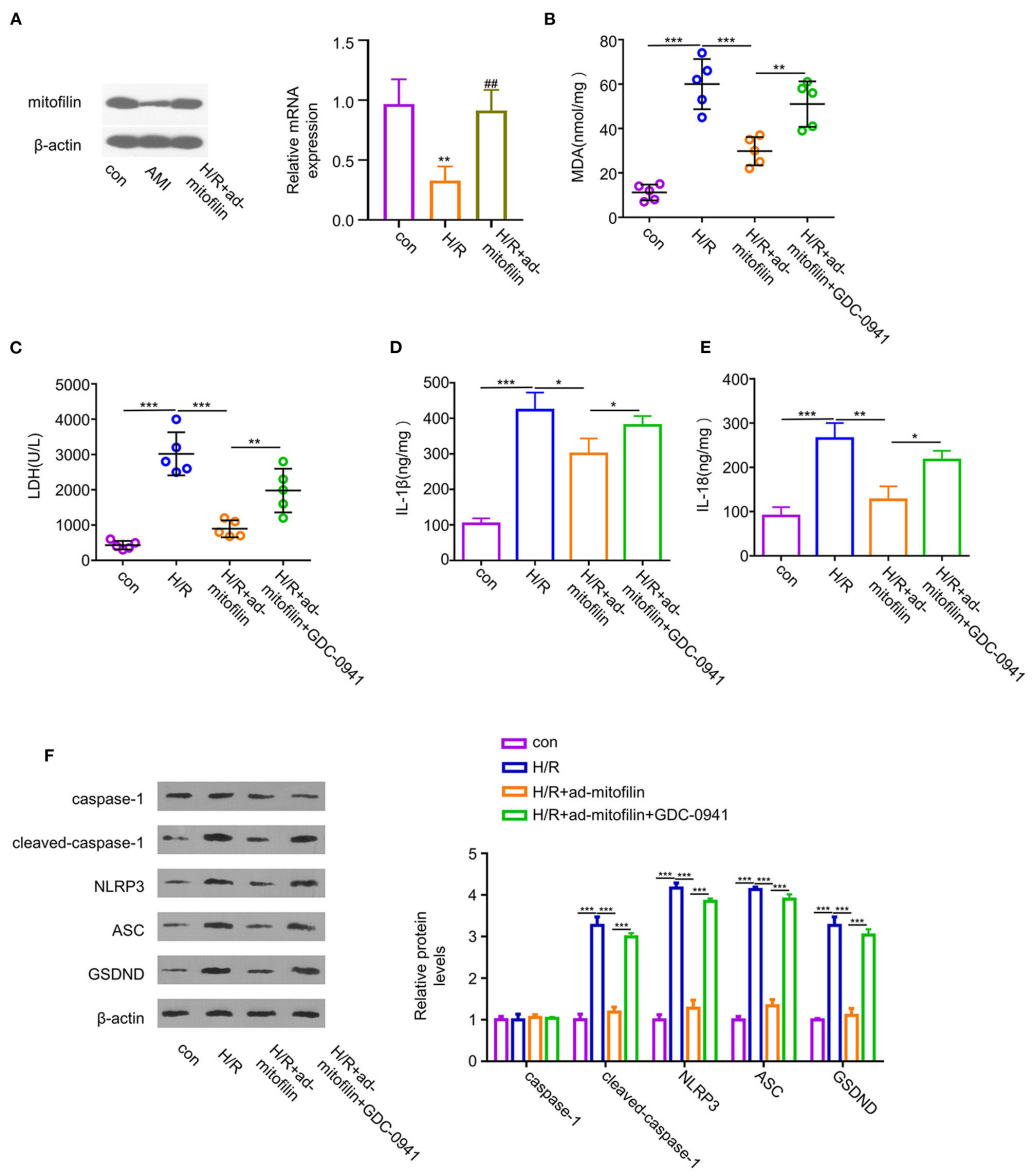
The protective effect of mitofilin in the H/R-induced H9C2 damage is dependent on PI3K activity. **(A)** Hypoxia/reoxygenation induced a decrease in the mitofilin expression level in H9C2 cells. The level of mitofilin protein was increased after mitofilin overexpression. **(B,C)** Hypoxia/reoxygenation induced increase of the MDA level and LDH level was partially reduced after mitofilin overexpression. The presence of GDC-0941 (a PI3K inhibitor) abrogated the rescue effect. **(D,E)** Hypoxia/reoxygenation induced the increase of IL-1β and IL-18 levels in H9C2 cells, and mitofilin overexpression reduced their levels. The presence of GDC-0941 abrogated the rescue effect. **(F)** Mitofilin overexpression suppressed the protein levels of cleaved-caspase-1, NLRP3, ASC, and GSDND in H9C2 cells upon H/R induction, and the effects were abrogated by GDC-0941. *P < 0.05, **P < 0.01, and ***P < 0.001. ##: H/R vs H/R+ad-mitofilin: *P* < 0.01.

To further validate that the anti-pyroptotic effect of mitofilin is dependent on the PI3K pathway, we examined the effect of mitofilin overexpression on H/R-induced pyroptosis in the presence of an effective PI3K inhibitor (GDC-0941). The overexpression of mitofilin in H9C2 cells upon H/R induction was confirmed by western blot and quantitative reverse transcription PCR (RT-qPCR) (Figure 5A). The levels of MDA, LDH, IL-1β, and IL-18 in H9C2 cells were increased after H/R induction, while levels were partially decreased after mitofilin overexpression (Figures 5B–E). The addition of a PI3K inhibitor largely abrogated the protective effect of mitofilin overexpression (Figures 5B–E). Additionally, the presence of PI3K inhibitor increased the protein levels of cleaved-caspase-1, NLRP3, ASC, and GSDND after mitofilin overexpression (Figure 5F). Together, these data suggest that the anti-pyroptotic effect of mitofilin is dependent on PI3K activity.

## Discussion

Pyroptosis, which is mediated by the inflammasome, is an inflammatory form of lytic programmed cell death frequently occurring upon infection with intracellular pathogens (17, 18). It has been reported that the pyroptosis of cardiomyocytes is an important contributor to MI progression (9). The morphological features of pyroptosis in cardiomyocytes include the formation of the inflammasome, the formation of pores in the plasma membrane, cell swelling, the rupture of membranes, and the release of inflammatory factors, such as IL-1β and IL-18 (19, 20). Our *in vivo* and *in vitro* experiments demonstrated that, upon AMI induction, cardiomyocytes displayed typical features of pyroptosis, such as the increased level of MDA, LDH, IL-1β and IL-18 and the loss of membrane integrity. Since pyroptosis is a programmed cell death mediated by inflammasome (8), we further examined the protein level of NLRP3, which has been widely recognized as a major factor in inducing pyroptosis (21). Once NLRP3 is activated, it promotes the activation of other pyrogenic factors to promote pyroptosis, such as caspase 1 (22, 23). In the present study, we showed that AMI induction in the mouse model and H/R induction in the cell model significantly increased the levels of NLRP3 and cleaved-caspase-1, as well as ASC and GSDND, which act as effectors in pyroptosis. Together, our data suggest that pyroptosis contributes to the cell death of cardiomyocytes in an AMI model.

To the best of our knowledge, this is the first report which states that mitofilin mitigates AMI-induced myocardial injury by regulating pyroptosis in cardiomyocytes. Mitofilin is an important component of the inner mitochondrial membrane (IMM), which plays a key role in controlling mitochondrial cristae morphology (24). A large number of studies have suggested the potential roles of mitofilin in cardiomyocyte function and injury. Mitofilin was first discovered as the “heart muscle protein” (25), and downregulating mitofilin in cardiomyocytes can induce apoptosis (12). However, the roles of mitofilin in regulating other forms of cell death in cardiomyocytes have not been evaluated. Interestingly, we found that mitofilin expression was reduced in the myocardial tissue and the cell model of AMI. Importantly, mitofilin overexpression by adenovirus administration effectively ameliorated AMI-induced pyroptosis in heart tissues. Moreover, silencing mitofilin in a cell model exacerbated pyroptosis upon AMI induction. Therefore, a high level of mitofilin expression in cardiomyocytes is required for the protection of pyroptosis upon AMI induction.

It is well-known that PI3K and its downstream signaling effectors (such as, the serine/threonine kinase AKT) are implicated in the pro-survival signals in different conditions (15, 26). For example, the activation of the PI3K/AKT pathway can suppress cell death in myocardial ischemic cells (27). Our study further showed that mitofilin knockdown could inhibit the activity of the PI3K/AKT pathway, while mitofilin overexpression promotes the activation of the PI3K/AKT pathway. Importantly, we further showed that the PI3K inhibitor largely abrogated the anti-pyroptotic effect of mitofilin overexpression. Therefore, these data suggest that mitofilin alleviates pyroptosis in AMI through the activation of the PI3K/AKT pathway. However, the detailed molecular mechanisms by which mitofilin activates the PI3K/AKT pathway warrant further investigation.

## Conclusion

In summary, our present studies provided evidence that mitofilin serves as an important protective factor to mitigate AMI-induced myocardial injury by inhibiting pyroptosis in cardiomyocytes. Our study also highlights the implication of the PI3K/AKT pathway in protecting pyroptosis in AMI. Future work needs to focus on the molecular mechanisms of how mitofilin activates the PI3K/AKT pathway.

## Data Availability Statement

The datasets presented in this study can be found in online repositories. The names of the repository/repositories and accession number(s) can be found in the article/supplementary material.

## Ethics Statement

The animal study was reviewed and approved by West China Hospital.

## Author Contributions

MM drafted the manuscript and collected data and algorithms. S-cL and K-yD were responsible for animal experiments and data analysis. QW was responsible for statistical analysis and cell culture. MM and YH were received grants. All authors provided approval for the final version of the manuscript.

## Funding

This work was supported by the Applied and fundamental study of Sichuan Province (No. 2017JY0026), the Fellowship of China Postdoctoral Science Foundation (No. 2020M683325), the Post-Doctor Research Project, West China Hospital, Sichuan University (No. 2020HXBH048), and the Innovative Scientific Research Project of Medical Youth in Sichuan Province (No. Q20061). Specifically, the funding was used for data analysis, the purchase of material consumed in the present study, publication costs, instrument use costs, etc. Additionally, the stipend of graduate students comes from the funding.

## Conflict of Interest

The authors declare that the research was conducted in the absence of any commercial or financial relationships that could be construed as a potential conflict of interest.

## Publisher's Note

All claims expressed in this article are solely those of the authors and do not necessarily represent those of their affiliated organizations, or those of the publisher, the editors and the reviewers. Any product that may be evaluated in this article, or claim that may be made by its manufacturer, is not guaranteed or endorsed by the publisher.
